# Congruent pattern of accessibility identifies minimal pore gate in a non-symmetric voltage-gated sodium channel

**DOI:** 10.1038/ncomms11608

**Published:** 2016-05-17

**Authors:** Kevin Oelstrom, Baron Chanda

**Affiliations:** 1 Department of Neuroscience, School of Medicine and Public Health, University of Wisconsin, 1111 Highland Avenue, Room 5505, WIMR Tower II, Madison, Wisconsin 53705, USA; 2 Department of Biomolecular Chemistry, School of Medicine and Public Health, 420 Henry Mall, Room 1135 Biochemistry Bldg, Madison, Wisconsin 53706, USA; 3 Molecular & Cellular Pharmacology Graduate Training Program, School of Medicine and Public Health, University of Wisconsin, Madison, Wisconsin 53705, USA

## Abstract

Opening and closing of the central ion-conducting pore in voltage-dependent ion channels is gated by changes in membrane potential. Although a gate residue in the eukaryotic voltage-gated sodium channel has been identified, the minimal molecular determinants of this gate region remain unknown. Here, by measuring the closed- and open-state reactivity of MTSET to substituted cysteines in all the pore-lining helices, we show that the state-dependent accessibility is delineated by four hydrophobic residues at homologous positions in each domain. Introduced cysteines above these sites do not react with intracellular MTSET while the channels are closed and yet are rapidly modified while the channels are open. These findings, in conjunction with state-dependent metal cross-bridging, support the notion that the gate residues in each of the four S6 segments of the eukaryotic sodium channel form an occlusion for ions in the closed state and are splayed open on activation.

## Abstract (web summary)

Gating of the central pore in voltage-dependent ion channels is mediated by changes in membrane potential. Here, the authors use substituted cysteine accessibility and metal cross-bridging to identify gate residues that form a physical occlusion when closed in a eukaryotic voltage-gated sodium channel.

## Graphical abstract

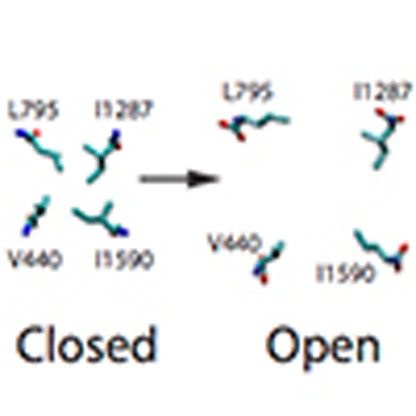

Voltage-gated ion channels are elementary molecular units that are responsible for electrical signalling that underlie nerve impulses, muscle contraction, release of neurotransmitters and in some instances transcriptional regulation. The voltage-gated sodium channel, which belongs to the voltage-gated ion channel superfamily, is a transmembrane protein that allows selective passage of sodium ions on depolarization^1^. The most prevalent eukaryotic sodium channels are functionally and structurally asymmetric although the overall architecture is similar to the symmetric voltage-gated potassium ion channels^2^. They are composed of four homologous domains arranged around a central ion-conducting pore, each domain consisting of six transmembrane helices (S1–S6) and one reentrant loop^3^,^4^. Upon an increase in membrane potential, the voltage-sensing modules of domains I–III activate rapidly, which leads to opening of the pore gates. This depolarization also drives the slow activation of voltage sensors of domain IV, which allows the open channels to subsequently inactivate^5^. Despite much progress in our understanding of gating in non-symmetric ion channels, the molecular determinants that define the minimal pore gate in these channels remain unknown.

Although not the case for every voltage-gated ion channel, the passage of ions through voltage-dependent calcium, potassium and sodium channels has been shown to be controlled by an intracellular gate near the lower S6 (refs 6, 7, 8, 9, 10, 11, 12). This conclusion is largely based on accessibility studies using the membrane impermeable thiol-modifying reagent 2-(trimethylammonium)ethyl methanethiosulfonate (MTSET). The observation of state-dependent accessibility of MTSET to introduced cysteine residues within the pore allows for the gate location to be determined. Sites that show a many fold increase in accessibility to MTSET when channels are open compared with closed are presumed to be inside the gate; those that can be modified when closed must be outside the gate. The residues found at the transition point are therefore by this definition the gate residues.

The activation gate within the Shaker potassium channel was shown to prevent ion permeation when closed by presumably acting as a steric barrier; there is simply not enough space for ions to enter or leave the pore when the gate is in its closed conformation^10^. This notion is supported by structures of both potassium channels and prokaryotic sodium channels, which clearly show that the intracellular pore gate can form an occlusion that will prevent transit of hydrated ions^13^,^14^,^15^,^16^,^17^. Owing to symmetry, the gate residues in each of these circumstances are identical, but that is not likely to be the case for eukaryotic voltage-gated calcium and sodium channels. Accessibility studies on eukaryotic voltage-gated calcium channels have identified sites inside the closed gate, but the residues that constitute the gate have not been identified^11^. Previously, we have systematically examined the accessibility of a series of residues in one of the pore-lining helices in the rat skeletal muscle sodium channel to identify the gate location^12^. We found that I1590 in the S6 of domain IV acts as a barrier that hinders MTSET access to the pore, suggesting that this residue may also contribute to the gating mechanism, which prevents ion conduction while the channel is closed.

To identify the other residues that define the minimal pore gate, here we examine the pattern of accessibility of the lower S6 segments of domains I–III in the skeletal muscle sodium channel. On the basis of our sequence alignment and previous accessibility studies, we identified analogous stretches in S6 of domains I–III for accessibility studies. Single cysteines were substituted in place of residues in this region and their accessibility to MTSET was determined while the channels were held closed or open. We find that a ring of bulky hydrophobic residues constitutes the most extracellular site that is able to react with intracellular MTSET while channels are closed. Paired cysteines introduced in this ring are able to form a state-dependent metal cross-bridge indicating that these four residues define the primary activation gate within a eukaryotic voltage-gated sodium channel.

## Results

### State-dependent accessibility of pore-lining cysteines

**Figure 1:**
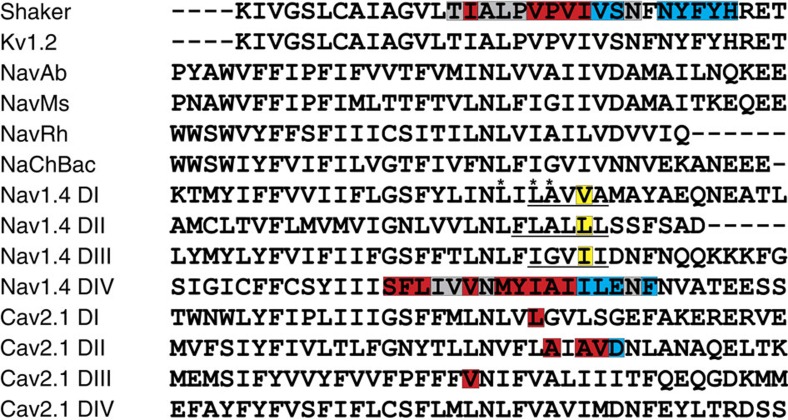
Pore-lining residues in S6. Amino-acid sequence alignment of the S6 segments from Shaker, K_v_1.2, four prokaryotic sodium channels, Na_v_1.4 and Ca_v_2.1. Red highlighting indicates sites that display state-dependent accessibility to MTSET and those marked in blue denote positions that can be modified while channels are closed. Grey highlighting indicates positions that show no response to MTSET treatment. Residues predicted to form the hydrophobic gate are outlined in yellow and those that are underlined were mutated to cysteine and used for accessibility experiments. Asterisks indicate the location of residues that are part of the WSW triple mutant.

To determine the residues from DI–DIII that form the sodium channel activation gate with DIV site I1590, we introduced 5–6 cysteine residues within the predicted gating region of each domain and examined the accessibility of intracellular MTSET to each of these sites (Fig. 1) (ref. 12). Normally, the sodium channel inactivation gate will occlude the pore after channel opening, hindering access to the pore by intracellular reagents such as MTSET^18^. We have circumvented this problem by creating all cysteine constructs in the background of the L435W/L437S/A438W triple mutant (WSW), which effectively removes fast inactivation and does not display any modification by MTSET (Fig. 1) (ref. 12). Using the WSW background, we can be confident that MTSET has unhindered access to the pore and any observed modification is only due to the reaction of MTSET with the inserted cysteine (Supplementary Fig. 1).

**Figure 2:**
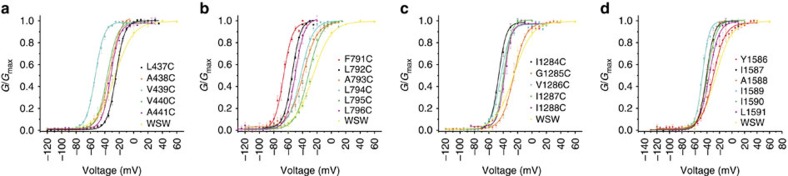
Conductance–voltage relationships (G–Vs) for DI–DIV S6 cysteine mutants. Normalized G–Vs for (**a**) DI, (**b**) DII mutants, (**c**) DIII and (**d**) DIV mutants were generated by measuring the peak tail currents elicited from inside-out patches containing mutant channels over a range of potentials. All patches were held at −120 mV and subjected to varying ranges of depolarizations depending on the particular mutant. Conductance values were normalized by dividing the peak tail current value at a given voltage by the maximum tail current value obtained and fit to a Boltzmann function to determine *V*_1/2_ and *Z* values (Table 1).

Before performing any modification experiments, we obtained conductance–voltage relationships (G–Vs) for each mutant by measuring tail currents in response to a series of depolarization steps from inside-out patches excised from *Xenopus* oocytes (Fig. 2). The voltage at which the conductance was half-maximal (*V*_1/2_) for the mutants tested in this study was in the range of −67 mV to −25 mV (Table 1). Not only is this information useful for understanding how the mutation affects gating but it also tells us what voltage to use while applying MTSET for open-state modification experiments (that is when apparent *P*_o_ is maximum). Likewise, we also assessed the time period required for recovery from slow inactivation (see Methods).

### V440 is the gate residue in domain I

**Figure 3:**
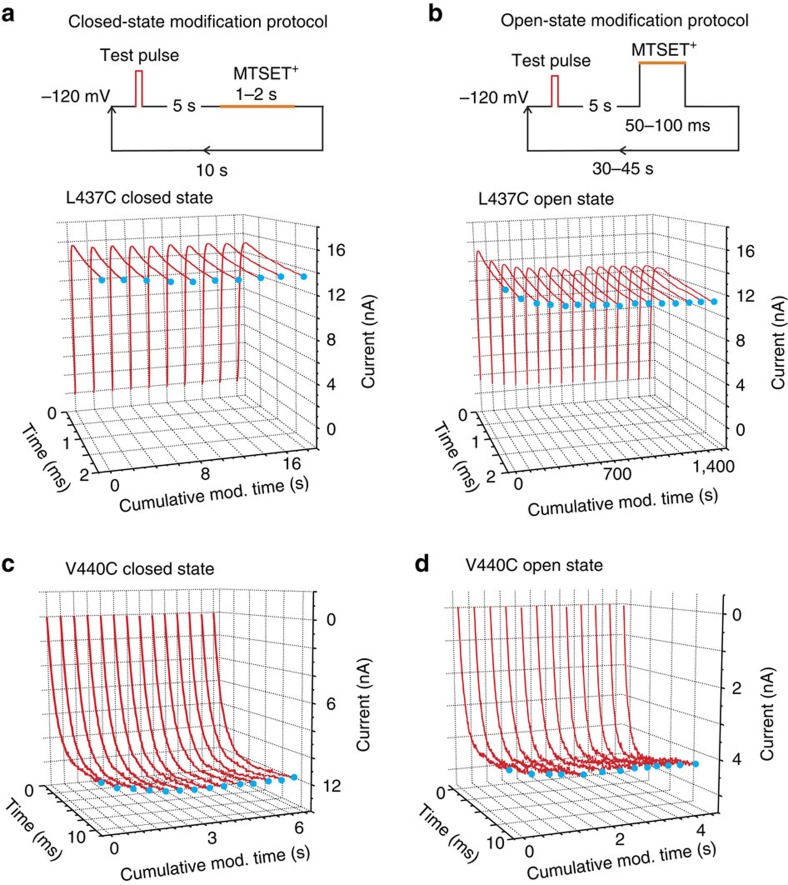
Reaction of intracellular MTSET with two cysteine residues within the DI S6. (**a**, top) Example MTSET perfusion and voltage protocol for a closed-state modification experiment. In this particular case, a 2-ms test pulse to 60 mV was used to evoke outward current; however, in some situations we used potentials to observe inward current. (**a**, bottom) Exemplar traces from a closed-state modification experiment using L437C channels. The concentration of MTSET was 1.4 mM. (**b**, top) Example MTSET perfusion and voltage protocol for an open-state modification experiment. Depending on the cysteine construct, different test pulse potentials were used to evoke either outward or inward current. (**b**, bottom) Exemplar traces from an open-state modification experiment using L437C channels. (**c**) Closed-state modification traces for V440C. (**d**) Open-state modification traces for V440C. The concentration of MTSET was 180 μM and the test pulse was −40 mV for 10 s.

On the basis of sequence homology (Fig. 1), we posited that V440 is part of the gate, or the ‘gating component' from domain I of the sodium channel. Therefore, sites more extracellular to V440 are expected to react with intracellularly applied MTSET only when the channels are held open while V440C and those below will be modified even when the channels are held closed. To validate this notion, we measured the accessibility of five introduced cysteines within DI to intracellularly applied MTSET. An example of how these experiments are performed, as well as the generated data, is provided in Fig. 3.

When a patch is excised from the membrane of a *Xenopus* oocyte expressing L437C channels, we can elicit outward current by applying a positive test pulse from a holding potential of −120 mV (Fig. 3a, red line at cumulative modification time point=0). Note that the duration and voltage of the test pulse is adjusted to elicit maximum currents while mitigating the accumulation of slow inactivated states. After returning to −120 mV and closing the channels, MTSET is rapidly perfused into the bath solution for 1–2 s and then washed out for 10 s. After MTSET washout, the same test pulse is applied and the measured current is plotted along the corresponding cumulative modification time point, in this case for 2 s. By repeating this process and tracking a change in a property of the current, in this case current amplitude, one can assess if MTSET reacts with L437C while the channel is closed. Since we do not see any modification in the currents, we know that L437C is not accessible to MTSET while the channel is closed (Fig. 3a).

A similar voltage and perfusion protocol was used to determine if MTSET can react with L437C while the channel is open, with a few exceptions. For the same patch containing L437C channels, the same test pulse is used, but this is followed by bath application of MTSET while the channel is held open (Fig. 3b) at voltages where the apparent open probability is maximum. After MTSET application, washout occurs for 30–45 s to ensure removal of MTSET and recovery from slow inactivation (see Methods). Repetition of this process reveals that the peak current of a patch containing L437C channels decreases after these channels have been exposed to MTSET while open (Fig. 3b). The fact that L437C channels display state-dependent accessibility to MTSET indicates that this position is protected from MTSET by the pore gate when channels are closed.

Using the same modification protocols for V440C, we see that even when the channels are held closed during MTSET exposure the peak current changes, in this case increasing over time (Fig. 3c). This was somewhat surprising initially because the positively charged-SET adduct on a pore-lining cysteine is expected to reduce the current. However, on examination of the kinetics, we realized that the addition of charge caused a shift in the gating behaviour as a result of which channels open at more hyperpolarized potentials and thus elicit larger currents during test pulses. In all other sites tested, we compared the current kinetics before and after modification to ensure that the MTSET modification is causing only current inhibition rather than modifying the gating properties.

When the open-state modification protocol was tested for V440C, we observed that the apparent modification rates were identical to those in the closed state (Fig. 3d). The fact that the cysteine at position 440 can react with intracellular MTSET while the channel is closed suggests that this site could be part of the gating mechanism, which regulates MTSET access to the pore. To test whether the sites above V440 could also fulfil this role, we performed accessibility measurements at these A438 and V439. Our data show that in contrast to V440, sites A438 and V439 do not react with cysteine-reactive MTSET when the channels are closed suggesting that V440 lies at the transition from sites that are only accessible when channels are open to those that can be modified even when channels are closed (data summarized in a later section).

To thoroughly assess the accessibility of MTSET to cysteine residues inserted in the lower DI S6, it was necessary to test cysteine accessibility at two positions that are part of the background WSW triple mutant (Fig. 1). When L/S437 is mutated to a cysteine, we observe that fast inactivation is removed and that modification at this site by MTSET is state-dependent (Fig. 3a,b). When A/W438 is replaced with a cysteine, we observe almost a complete return of fast inactivation and this effect is reversed after MTSET is applied to open A/W438C channels (Supplementary Fig. 2). In addition, it appears that the MTSET modification also causes a decrease in the total current because the amplitude of the fully modified current is less than that of the peak current before modification. Thus, modification of this site has a dual effect on gating and conductance. Nonetheless, the rate of modification can be quantified by measuring the current at the end of the test pulse when the channels are in steady state. In the closed state, we did not observe any appreciable modification of this site. Similarly, V439C did not show any closed-state modification but was rapidly modified in the open state (data summarized in a later section).

When we tested open- and closed-state modification rates of V441C, we observed that those rates were identical suggesting that this site is definitely below the channel gate. Thus, in domain I, the V440C residue is part of the gate that delimits the accessibility to the open pore.

### Leucine 795 is the gating component for domain II

**Figure 4:**
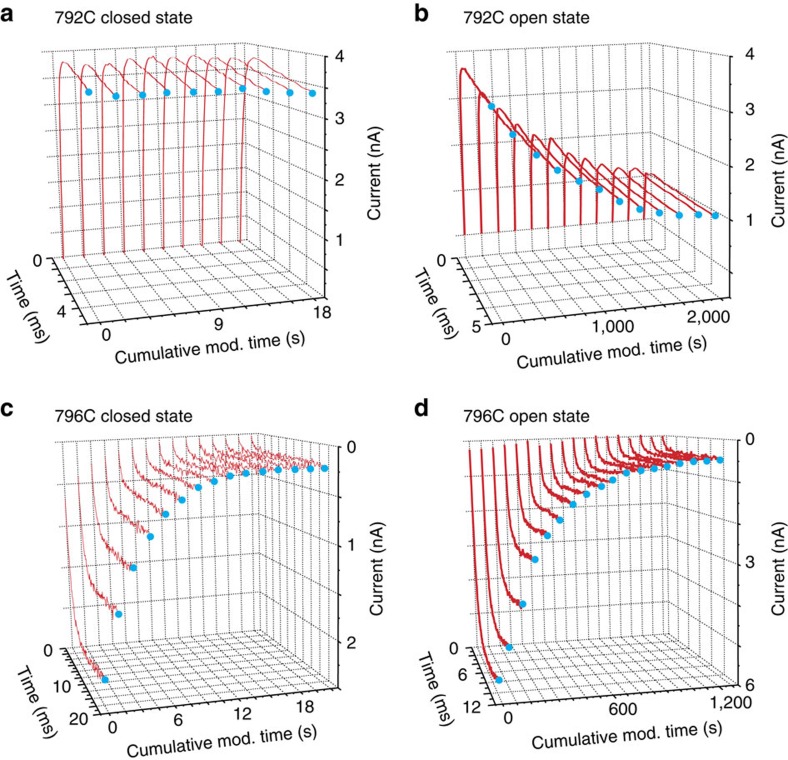
Accessibility experiments for two cysteine residues within the DII S6. (**a**) Closed-state modification experiment for L792C using 5-ms test pulses to 60 mV. (**b**) Open-state modification experiment for L792C. The concentration of MTSET was 744 μM (**c**) Closed-state modification traces for L796C using 20-ms test pulses to −30 mV. The concentration of MTSET was 1.3 mM. (**d**) Open-state modification traces for L796C using 12-ms test pulses to −30 mV. The concentration of MTSET was 1.8 mM.

To determine if the DII S6 regulates sodium channel gating in the same manner as DI and DIV, we observed the changes in modification rates for six introduced cysteines to intracellular MTSET. L792C, a site predicted to be above the gate, displays a change in peak current only after open channels are exposed to MTSET (Fig. 4a,b). L796C, a site expected to be below the gate, shows a significant decrease in sodium current after MTSET application to closed channels (Fig. 4c,d). We see that F791C and A793C do not react with MTSET while each is closed, but show a large change in accessibility after MTSET application to open channels (data summarized in a later section). Peak currents for L794C channels did not change, regardless of the channel state while MTSET was present. This is could either mean that the cysteine at that position is not reacting to intracellular MTSET or that on reaction it has no functional effect on channel conductance. We favour the interpretation that the cysteines at these positions are unable to react with MTSET since we find it improbable that a charged adduct like MTSET would have no effect on transport of cations especially when they are likely to be in close proximity. Nonetheless, our assay here is not designed to discriminate between either of these two possibilities.

Like L796C, L795C channels were significantly modified while the channels were closed and open. The fact that L795C is the most extracellular site in DII to react with MTSET while channels are held closed suggests that L795 is the gating component for DII.

### Isoleucine 1287 of domain III is part of the gate

**Figure 5:**
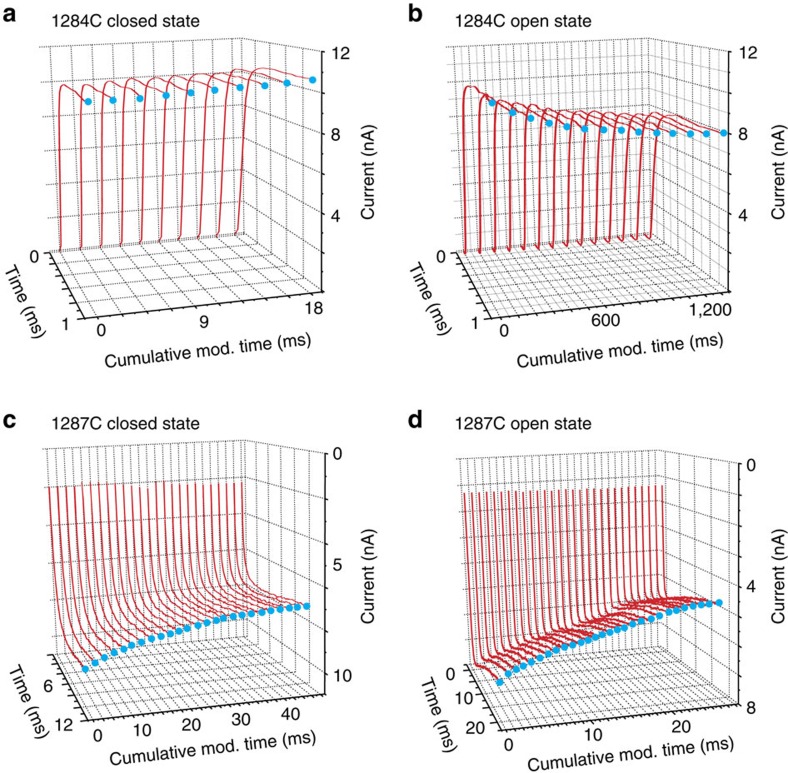
Modification time course of two cysteine residues within the DIII S6. (**a**) Exemplar traces from a closed-state modification experiment using I1284C channels. (**b**) Exemplar traces from an open-state modification experiment using I1284C channels. One-millisecond test pulses to 60 mV were used. (**c**) Time course of the reaction of MTSET with I1287C while channels are held closed. The concentration of MTSET was 19.6 mM and test pulses were at −30 mV for 12 ms. (**d**) Open-state modification traces for I1287C. The concentration of MTSET was 100 μM and test pulses were at −30 mV for 20 ms.

To define the DIII residue involved in channel gating, we measured the modification rates for five cysteine residues introduced into the lower DIII S6. Subsequent test pulses after application of bath MTSET to closed I1284C channels reveals no change in peak current, however, test pulses after open channel exposure show that this site can be modified (Fig. 5a,b). G1285C displays similar behaviour, while V1286C, like L794C of DII, does not show any change in current amplitude after MTSET application, regardless of channel state (data summarized in a later section). In Fig. 5c,d, we see that I1287C reacts with MTSET when the channels are closed and open. Like I1287C, I1288C is also modified when the channels are closed. I1287 is found at the transition between sites that can only react with MTSET when channels are open and those that can even when closed, indicating that this position contributes to the gating machinery, which allows passage of sodium ions through the pore after channel activation.

### Four hydrophobic residues regulate gating

**Figure 6:**
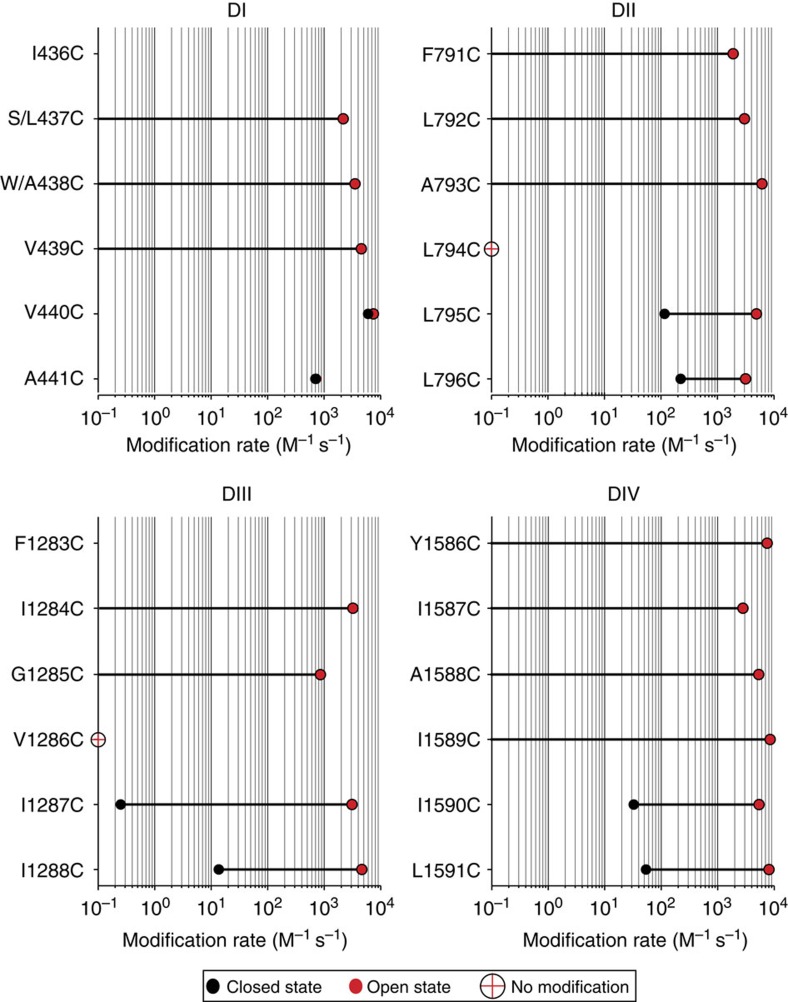
State-dependent accessibility of lower DI–DIV S6 cysteine mutants. Cysteine modification rates in the closed (filled black circles) and open (filled red circles) states are plotted on a logarithmic scale for 5–6 positions within each domain. Each point is the mean of at least three experiments; standard errors are smaller than symbols. The length of the bar represents the fold change in rate between the closed and open states. Sites above V440C, L795C, I1287C and I1590C have closed-state reactivity values of <1 M^−1^ s^−1^. L794C and V1286C displayed no change in peak current after MTSET exposure (red cross filled circles). Depending on the rates of modification, the concentration of MTSET used to test each site ranged from 100 μM to 20 mM.

**Figure 7:**
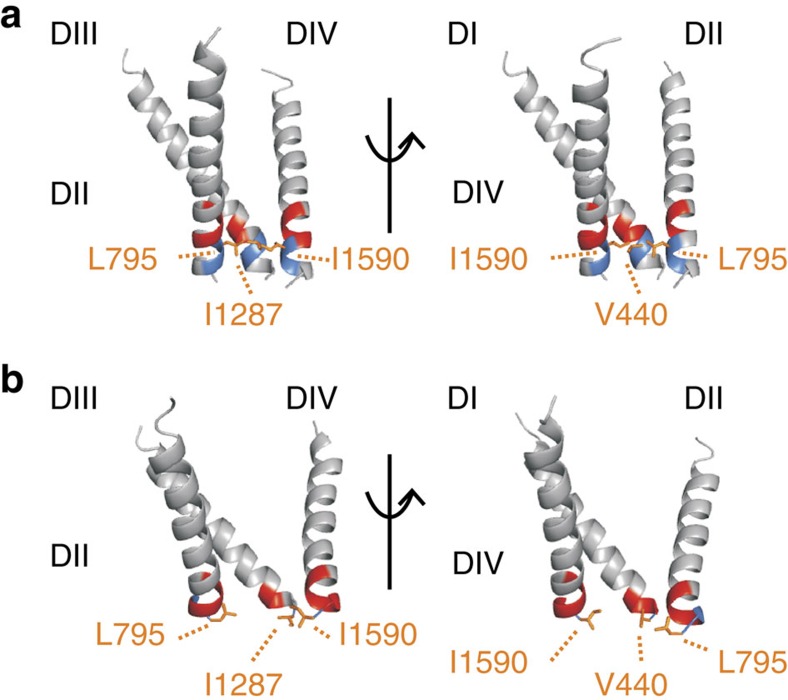
Accessibility data mapped onto structural data. (**a**) Closed-state homology model showing only DII–DIV (left) and only DI, DII and DIV (right). Hydrophobic gating residues (orange) are found at the transition from sites that can only react with MTSET when the channel is open (red) to sites that can be modified even when the channel is closed (blue). (**b**) The same data represented on an open-state homology model showing only DII–DIV (left) and only DI, DII and DIV (right) highlights how movement of the hydrophobic gating residues on channel activation allows for ionic conduction.

Our data reveal a distinct point in all four domains separating sites exhibiting state-dependent and state-independent reaction with MTSET. Sites above V440C, L795C, I1287C and I1590C show modification rates <1 M^−1^ s^−1^ in the closed state, but these increase by at least 10,000-fold in the open state (Fig. 6). The large increase in reactivity on channel opening implies that these sites are protected from MTSET modification while channels are closed, presumably by the gate. At or below these positions, we see significant modification in both closed and open states. A more than 10,000-fold increase in closed-state accessibility at these sites compared with state-dependent sites indicates that these cysteines are no longer protected by the gate. After systematically observing the state-dependent accessibility of intracellular MTSET to cysteines introduced within the lower S6 of DI–DIV of a voltage-gated sodium channel, it is clear that the gating component from each domain is a hydrophobic residue at the same position (Fig. 7a,b). Thus, much like the voltage-gated Shaker potassium channel, an intracellular gate composed of four hydrophobic residues regulates MTSET access to the pore of a eukaryotic voltage-gated sodium channel.

### Gated access of small cations to the pore

To clearly demonstrate that residues V440, L795, I1287 and I1590 form the activation gate in sodium channels, which regulates the passage of not only MTSET but also sodium ions through the channel pore, smaller cysteine reactive probes like Cd^2+^, Zn^2+^ and Ag^+^ can be employed to act as surrogates for sodium ions. MTSET is much larger than these four ions, so it certainly is a possibility that V440, L795, I1287 and I1590 only prevent MTSET from getting into the pore, but not necessarily sodium. Cadmium and silver were able to modify a pore-lining site in the Shaker potassium channel in a state-dependent manner, indicating that potassium permeation is regulated by the same intracellular gate as MTSET^9^,^10^. We attempted to address this issue by assessing the accessibility of Cd^2+^ to a cysteine above the gate in DIV of the sodium channel. However, we observed that the WSW background itself displayed a change in current properties on intracellular application of 10 μM Cd^2+^. Similarly, 10 nM free Ag^+^ reduced sodium current in WSW by 25%. There are four additional intracellular cysteines that were not mutated in the sodium channel and it is likely that the Cd^2+^ and Ag^+^ react with one or more of these cysteines. As stated earlier, MTSET does not show any reactivity to these cysteines and previous attempts to mutate these cysteines to create a cysteine-less background resulted in non-functional protein.

**Figure 8:**
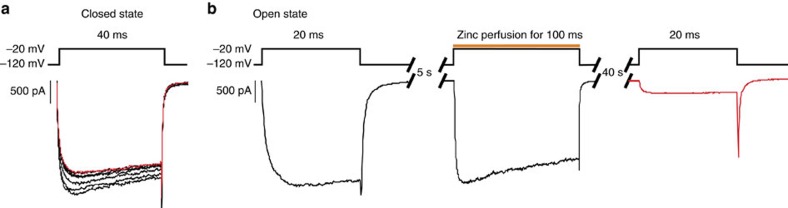
Zinc accessibility experiments with V440C–I1287C channels. (**a**) Inward sodium current from V440C–I1287C channels in response to 40-ms test pulses to −20 mV was recorded before and after multiple treatments of 10 μM zinc for 2 s each while the channels were held closed. Ten seconds elapsed between each test pulse. The red trace indicates the final test pulse after the cumulative exposure time. The decrease in current is comparable to that seen for WSW in both closed- and open-state experiments. (**b**) The effect of zinc application onto open channels was assessed by giving a test pulse to −20 mV for 20 ms before zinc treatment (black trace), a 5-s recovery at −120 mV, a 100-ms exposure to 10 μM zinc while the channels were held open at −20 mV (orange line), a 40-s washout and recovery period at −120 mV, followed by another test pulse to −20 mV for 20 ms (red trace). No further decrease in current is observed after a second round of zinc treatment. Sodium current recorded during the first exposure to zinc shows no sign of inhibition, suggesting that zinc does not form a bridge with V440C and I1287C while channels are open, but does so after entering the pore and channel closure. The data are representative of three separate experiments.

Interestingly, 10 μM intracellular Zn^2+^ produces a minimal effect on sodium currents elicited from WSW channels. We took advantage of this situation by creating three mutants containing either four cysteines at V440, L795, I1287 and I1590, or pairs of cysteines at V440 and I1287 or L795 and I1590, and assessing their ability to form a bridge with zinc. Not only would zinc bridge formation demonstrate that the residues in question are in close proximity but it may also serve as a gating probe like Cd^2+^ and Ag^+^ if bridge formation is state-dependent. Of the three constructs, only V440C–I1287C expressed sufficient currents for study. When 10 μM Zn^2+^ was applied to the cytoplasmic side of patches expressing these channels while they were held closed, we were able to observe a very small decrease in sodium current, indicating that zinc is unable to reach the binding site effectively while the channel is closed (Fig. 8a). When zinc was applied to open channels, we did not see a decrease in current during the exposure period (Fig. 8b). If zinc were to form a cross-bridge with the cysteines in the open channel, we would have observed an immediate decrease in current on zinc application to the open channel. Strikingly, current inhibition is not observed until after the channel has been closed and then reopened during the subsequent test pulse (Fig. 8b). Note that any free zinc is washed when the channels are held closed. Our interpretation of these results is that zinc is unable to access the cysteines when the channel is closed, enters the inner vestibule upon channel opening and gets trapped when the channel gates are closed. Inhibition is observed in subsequent pulse because the trapped zinc ion is able to form a metal bridge with the two sites that must be coming together in close proximity in the closed state. We did not thoroughly test the kinetics of this metal bridge formation but our data suggest that this must be relatively rapid since no further inhibition is observed after the first current decrease. This behaviour highlights two points; zinc, like MTSET, displays state-dependent access to the pore, and V440C and I1287C must be in close proximity while the channel is closed to allow bridge formation. This sort of sequential state-dependence of zinc reactivity is rather unique to the best of our knowledge and establishes that these residues are components of the intracellular gate responsible for controlling the passage of sodium ions through the channel pore.

### Asymmetric contribution to the gating mechanism

While our findings support the original notion that the residues at paralogous positions in all four domains contribute to the pore gate, it is also clear from our functional data that some aspects of the structure in this region are distinctly asymmetric. If the structure were strictly symmetrical, then MTSET would have produced identical effects on equivalent sites. Examination of the rate of reactivity of the sites tested here (Fig. 6) and reported previously reveals an interesting aspect about our accessibility data^12^. The rate of reactivity of residues, which are below the gate in the closed state, increases as we move away from the gate to residues, which are more intracellular (Fig. 6). This would suggest that even in the closed state, the S6 helices are likely to be slightly bent, which would result in narrowing of the passageway leading up to the hydrophobic gate. In domain I, however, there is no difference between the closed- and open-state accessibility of the residues below the gate, which would imply that the S6 segment of DI does not undergo significant conformational change on channel opening. State-dependent accessibility rates of additional sites below the gate has to be tested in domain I to confirm this idea.

**Figure 9:**
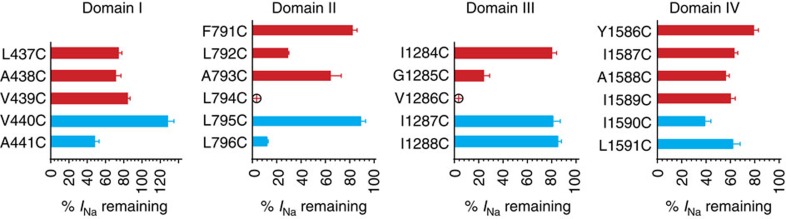
Change in current amplitude after MTSET treatment. By plotting the current amplitude after complete MTSET modification by the initial amplitude, the extent of current inhibition induced by MTSET can be visualized for DI–DIV cysteine mutants and compared with the WSW background. Except for V440C, L794C and V1286C, all mutants display a reduction in current after MTSET application. The current amplitude increases for V440C, while L794C and V1286C (red crosses) are either inaccessible to MTSET or their reaction with MTSET produces no functional change in current properties. Error bars represent s.e.m. of at least three independent experiments.

In addition to variability in the state-dependent accessibility rates, we also observe wide variations in the extent of current inhibition in sites at equivalent positions (Fig. 9). For instance, both L796C in domain II and A441C in domain I profoundly inhibit sodium conductance when they are modified by MTSET compared with I1288C and L1591C, which are in domains III and IV, respectively. The percent of sodium current that remains after complete MTSET modification reports on the extent to which the altered cysteine affects the ability of sodium ions to pass through the pore, be it through steric hindrance or charge repulsion, and this can only be assessed for the open state. Thus, our data on extent of current inhibition at equivalent positions in the sodium channel would suggest that at the very minimum when the channel is open the structure is asymmetric and the sites 796C and A441C are closer to the permeation pathway than equivalent positions in domains III and IV. This behaviour is observed not only in a non-symmetric sodium channel but also in a eukaryotic calcium channel, highlighting that these types of channels have a much more unique gating mechanism than previously anticipated^19^. Therefore, it appears that while voltage-gated calcium, potassium and sodium channels share a common feature for regulating ionic conduction by an intracellular gate, the structure of the open gate in these channel families may be quite different.

## Discussion

Given the rapid strides being made in structural biology, it appears imminent that the structures of a eukaryotic voltage-gated sodium channel will become available in the next few years^13^,^20^. These structures are likely to provide unprecedented details regarding the nature of the pore gate and give new insights into how this process is controlled^14^,^15^,^17^,^21^,^22^,^23^. Nonetheless, functional data, which includes accessibility measurements, will be critical to interpret these structures^24^. In the past, there have been instances where initial intuition based on the structure did not stand the scrutiny of subsequent functional or computational studies. A notable example is the MscS structure, which when first solved, was thought to be in an open state^25^. Subsequent studies by Sukharev and colleagues showed that while the pore gates in this structure were in a sterically open conformation, this region was too hydrophobic to allow water molecules from traversing through the apparently ‘open' channel^26^. Thus, it is essential to interpret structural data in light of functional and other biophysical measurements to gain a comprehensive understanding.

The notion that the gate of voltage-gated ion channels is in the intracellular side came from classic studies carried out by Armstrong on native voltage-gated potassium channels in the squid giant axon^27^,^28^,^29^. In the case of the voltage-gated sodium channels, the strongest early evidence of such a gate came from studies of local anaesthetic derivatives that showed classic open channel block^30^. This notion of an intracellular gate in the sodium channel was solidified by the remarkable discovery of resurgent sodium currents in Purkinje neurons^31^. Raman and Bean showed that an intracellular blocking particle (a portion of the beta 4 subunit) prevents the gate from closing and when it comes off, sodium ions can flux before the channels have the opportunity to close^32^. Under physiological conditions, this mechanism allows the neurons to fire repetitively and at a much higher frequency.

To date, the most rigorous way to determine the gate location is by measuring the accessibility of substituted cysteines in the pore-lining helices to polar MTS reagents or ions. Studies by Yellen and colleagues in the Shaker potassium channel have convincingly established that a ring of hydrophobic residues creates the gate in these channels^9^,^10^. However, similar studies on the BK channel show that the same region does not form a gate and the residues much further up in the S6 helix can react to polar MTS groups at a significant rate even when the channels are closed^7^. Substituted cysteine accessibility studies in an inactivation deficient version of the eukaryotic voltage-gated sodium channel established the location of the gate position in the S6 of domain IV (ref. 12). However, unlike tetrameric potassium channels, eukaryotic sodium channels lack exact symmetry and, therefore, the identity of the other constituents of this intracellular gate remain unclear.

In this report, by observing the state-dependent ability of intracellular MTSET to react with cysteines introduced into the lower S6 of a voltage-gated sodium channel, we show that paralogous positions within each domain demarcate a transition from sites only accessible while channels are open to those that can be modified in the closed channel (Fig. 7a,b). Positions 437–439 of DI, 791–793 of DII, 1,284–1,285 of DIII and 1,578–1,589 of DIV display no measureable reactivity to intracellularly applied MTSET when the channels are closed at −120 mV, indicating that these sites are not accessible in the closed state. When the channel is opened, these sites react to the same MTSET application with at least a 10,000-fold increase in reactivity. In contrast, cysteines inserted at positions 440–441 of DI, 795–796 of DII, 1,287–1,288 of DIII and 1,590–1,594 of DIV can be modified even when channels are closed^12^. Furthermore, using metal cross-bridge strategy, we were able to demonstrate that the gate residues on helices I and III are in close proximity in the closed state and move away from each other in the open state. (Fig. 8a,b).

The open channel probability for wild-type sodium channel has been estimated to be 10^−7^ at −80 mV (ref. 33). We applied MTSET at −120 mV for closed-state modification protocols to ensure that the channel open probability is even lesser than those at −80 mV. On the basis of the assumption that the channel gating is strictly coupled to pore opening, we estimate that the channel opening probability is few orders of magnitude smaller and this is likely to mitigate any effects of the mutations. Also note that our cysteine mutations are at single site unlike those in symmetric channels and therefore the effect of these mutations on gating is much less than that observed for potassium channels. Although some positions show up to 10,000-fold change in accessibility, we assign these residues as below the gate since we would expect even greater change in accessibility based on the changes on *P*_o_ values. Therefore, our criteria for defining the gate positions were not limited to observing large state-dependent changes in accessibility, but making sure that there was no observable reactivity in the closed state to the sites above the gate. Significant changes in state-dependent changes in accessibility could arise when the site undergoes a conformational change that reduces accessibility to charged group. For instance, a residue in a transmembrane helix may be buried in the lipid bilayer in a particular state, which would reduce its state-dependent reactivity. However, if it is placed behind a physical occlusion, this residue is unlikely to react at all. Therefore, rather than changes in state-dependent accessibility it is the lack of reactivity in the closed state that defines the gate positions in our measurements. Furthermore, in each of the domains, we identified multiple contiguous sites above the gate, which did not react in the closed state to MTSET suggesting that these positions were behind a physical occlusion in the closed state. A simple helical rotation, on the other hand, would be expected to expose some sites and bury others.

Although V440, L795, I1287 and I1590 contribute to forming the channel gate, the extent to which each residue contributes is not identical. Given that these channels are pseudo-symmetric, it is not surprising that there are differences in the degree of current inhibition at equivalent positions. For example, the current remaining for A441C (47.7%), L796C (11.6%), I1288C (85.0%) and I1591C (62.1%) is reduced to varying degrees by MTSET modification. These differences in current inhibition for paralogous sites must reflect the asymmetry in the open structure since we are able to measure currents only when the channels are open. Furthermore, a comparison of the rates of accessibility in the open and the closed state shows that the residues below the gate in domain I is unlikely to undergo a large change in accessibility on channel opening, which would suggest that this part of the S6 helix may not undergo a large-scale conformational change. This is striking because the domain I pore module is adjacent to domain IV voltage-sensing module that is known to behave differently than the other voltage-sensing domains of the sodium channel^2^. These observations raise intriguing questions about the sodium channel conformations and highlight the need for structure of eukaryotic sodium channels in conjunction with more extensive functional studies to map the gating motions of this region.

Taken together with previous studies, our findings described here suggest that the molecular mechanism that regulates Na^+^ conduction through voltage-dependent sodium channels involves an intracellular gate that is composed minimally of the four hydrophobic residues V440, L795, I1287 and I1590, which physically occlude the pore at hyperpolarized potentials. We do not have any information regarding the dynamics or structure of the pore gate, but based on homology models of existing prokaryotic bacterial sodium channels^12^,^14^,^15^,^17^, we can posit that these four residues will be sufficient to gate the access of hydrated ions through the pore. Also, based on sequence conservation, we propose that this ring of hydrophobic residues is likely to be the primary gate in all eukaryotic sodium channels. Nonetheless, we cannot rule out the possibility that there are secondary gates in other isoforms that may also aid in regulating the access to the pore^17^,^23^. Ultimately our results, in conjunction with emerging structural information, will allow us to precisely define the structure of the pore gate and give insights into motions that underlie pore gating during channel activation.

## Methods

### Molecular biology

Single cysteines were created using Quikchange mutagenesis (Stratagene, CA) and confirmed by gene sequencing. Forward primers: W/A438C, 5′- GGATCTCCTGCGTGGTGGC-CATGGCG -3′; V349C, 5′- CCTGGTGCGTGGCCATGGCGTACG -3′; V440C, 5′- GGGTGTGCGCCA-TGGCGTACGCTGAG -3′; A441C, 5′- GTGGTGTGCATGGCGTACGCTGAGCAG -3′; F791C, 5′- CTGTGCCTGGCTCTCCTGCTGAGTTCC -3′; L792C, 5′- GTTCTGCGCTCTCCTGCTGAGTTCC -3′; A793C, 5′- GTTCCTGTGCCTCCTGCTGAGTTCCTTC -3′; L794C, 5′- GGCTTGCCTGCTGAGTTCCTTCAGTGC -3′; L795C, 5′- GGCTCTCTGCCTGAGTTCCTTCAGTGCTG -3′; L796C, 5′- CCTGTGCAGTTCCTTCA-GTGCTGACAGCC -3′; I1284C, 5′- CCTCTTCTGTGGTGTCATCATCGACAACTTC -3′; G1285C, 5′- CATCTGCGTCATCATCGACAACTTCAACC -3′; V1286C, 5′- CGGTTGCATCATCGACAACTTCAACCAA-CAG -3′; I1287C, 5′- GGTGTCTGCATCGACAACTTCAACCAACAG -3′; and I1288C, 5′- GTCATCTGC-GACAACTTCAACCAACAGAAG -3′. Reverse primers: W/A438C, 5′- GCCACCACGCAGGAGATCCA-GTTGATGAGG -3′; V349C, 5′- GGCCACGCACCAGGAGATCCAGTTG -3′; V440C, 5′- CCATGGCGCAC-ACCCAGGAGATCCAG -3′; A441C, 5′- CGCCATGCACACCACCCAGGAGATCCAG -3′; F791C, 5′- GCC-AGGCACAGATTCAGGACCACCAGG -3′; L792C, 5′- GGAGAGCGCAGAACAGATTCAGG -3′; A793C, 5′- GCAGGAGGCACAGGAACAGATTCAGGAC -3′; L794C, 5′- CAGCAGGCAAGCCAGGAACAGATTCAGG -3′; L795C, 5′- CTCAGGCAGAGAGCCAGGAACAGATTCAGG -3′; L796C, 5′- GGAACTGCACAGG-AGAGCCAGGAACAG -3′; I1284C, 5′- CACCACAGAAGAGGTTGAGGGTGAAGAAG -3′; G1285C, 5′- GATGACGCAGATGAAGAGGTTGAGGGTG -3′; V1286C, 5′- GATGATGCAACCGATGAAGAGGTTGA-GGG -3′; I1287C, 5′- GTCGATGCAGACACCGATGAAGAGGTTGAGG -3′; and I1288C, 5′- GTTG-TCGCAGATGACACCGATGAAGAGGTTG -3′. Using *MfeI* and *AvrII* for DI, *AvrII* and *AatII* for DII, and *AatII* and *BspEI* for DIII, mutations were subcloned into the previously described fast inactivation removed WSW-Nav1.4 background^12^. For expression, constructs were linearized by NotI digestion and transcribed *in vitro* using the T7 RNA polymerase kit (Ambion Inc., CA).

### Oocyte expression

Oocytes were either purchased from Ecocyte Biosciences (Austin, TX) or were surgically obtained from adult *Xenopus laevis* under anaesthesia, consistent with the protocol approved by the Animal Care and Use Committee at University of Wisconsin-Madison. Stage V–VI oocytes were prepared by treatment with 1 mg ml^−1^ collagenase A (Roche) in a calcium-free ND96 solution for 1.0–1.5 h with shaking and then stored in calcium and BSA containing ND96 solution. ND96 solution: 96 mM NaCl; 2.5 mM KCl; 1 mM MgCl2; 5 mM HEPES; 1.8 mM CaCl2; 100 U ml^−1^ penicillin; and 100 g ml^−1^ streptomycin (pH 7.5). α-subunit and β_1_-subunit cRNA were co-injected into defoliculated oocytes in a 1:1 ratio of 2 μg μl^−1^ α-subunit and 400 ng μl^−1^ β_1_-subunit. Injected oocytes were maintained in ND96 solution in an 18 °C incubator and used for recordings 3–5 days after injection.

### Electrophysiology

Ionic currents were measured from inside-out patches excised from oocytes 3–5 days after injection. Standard solutions contained, in mM: NaF 100, KCl 20, EGTA 2, HEPES 10, MgCl_2_ 2.5, at pH 7.2 (bath); NaCl 115, KCl 5, CaCl_2_ 2, HEPES 10, at pH 7.2 (pipette). Typical pipette resistances were 500 kΩ–1.5 MΩ. On inside-out patch excision, currents were allowed to stabilize at −120 mV for 5 min. Electrophysiology recordings were acquired using Axopatch 1D controlled by pClamp (Molecular Devices, CA). Data were analyzed using Clampfit (Molecular Devices, CA), Origin (Originlab, MA) and Excel (Microsoft, WA).

### Chemical modification

A stock solution of MTSET (Toronto Research Chemicals, Ontario) was prepared daily and kept on ice. This stock solution was diluted to 100 μM–20 mM in bath solution and used immediately (within 1 min). Inside-out patches containing mutant channels were exposed to MTSET via a rapid perfusion system in which a computer-controlled valve was used to switch between standard bath solution and one containing MTSET 34,35. By switching between our standard bath solution and one of a lower ionic strength, it is possible to assess the dead time of the perfusion apparatus (typically 150 ms). This time was determined for each set of experiments and was accounted for to synchronize it with voltage pulses that open and close the channel. This method ensures that the reported reaction rates take into account only the time during which MTSET was present. For each experiment, the time constant of MTSET modification was calculated by plotting the cumulative exposure time to MTSET versus peak sodium current. The accessibility, or the apparent second-order rate constants, of MTSET to each cysteine mutant was determined by dividing the reciprocal of the time constant by the concentration of MTSET. For sites that required long exposure times, we took into account the fact that MTSET hydrolyses rather quickly by making an identical MTSET working solution in a separate perfusion line several minutes into the use of the initial working solution. By doing this, we were able to seamlessly add fresh MTSET containing solution to our experimental set-up and rule out the possibility of MTSET hydrolysis.

After prolonged depolarizations, it has been observed that fast inactivation-deficient preparations of sodium channels display an increased propensity to enter into a slow-inactivated state^36^. This trend was also noticeable for each mutant used in our experiments. To make sure that test pulse sodium currents represent decay owing to MTSET modification and not forms of slow inactivation, we used mock open-state protocols in which bath solution and not MTSET was applied to determine the necessary time between each exposure sweep. This guaranteed that each successive test pulse reported inhibition of current owing to MTSET modification and not slow inactivation.

## Additional information

**How to cite this article**: Oelstrom, K. and Chanda B.. Congruent pattern of accessibility identifies minimal pore gate in a nonsymmetric voltage-gated sodium channel. *Nat. Commun.* 7:11608 doi: 10.1038/ncomms11608 (2016).

## Supplementary Material

Supplementary Information Supplementary Figures 1-2

## Figures and Tables

**Table 1:**
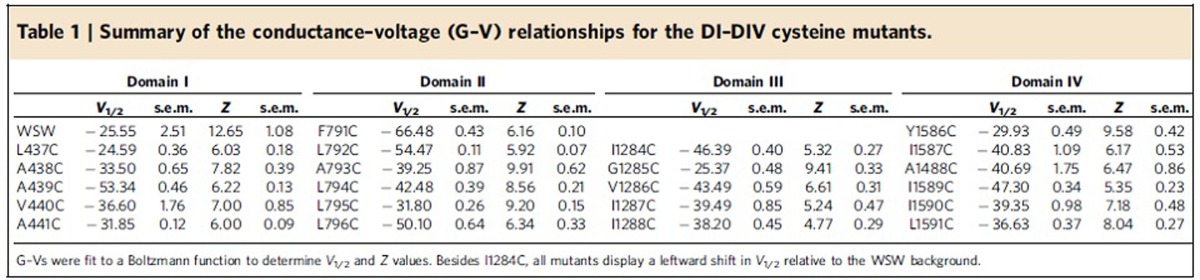
Summary of the conductance–voltage (G–V) relationships for the DI–DIV cysteine mutants.
