# Supplementary material for: Congruent pattern of accessibility identifies minimal pore gate in a non-symmetric voltage-gated sodium channel
Source: Nat Commun. 2016 May 17;7:11608. doi: 10.1038/ncomms11608 (PMC4873679; doi:10.1038/ncomms11608)
Supplement: Supplementary Information — Supplementary Figures 1-2 [file ncomms11608-s1.pdf]

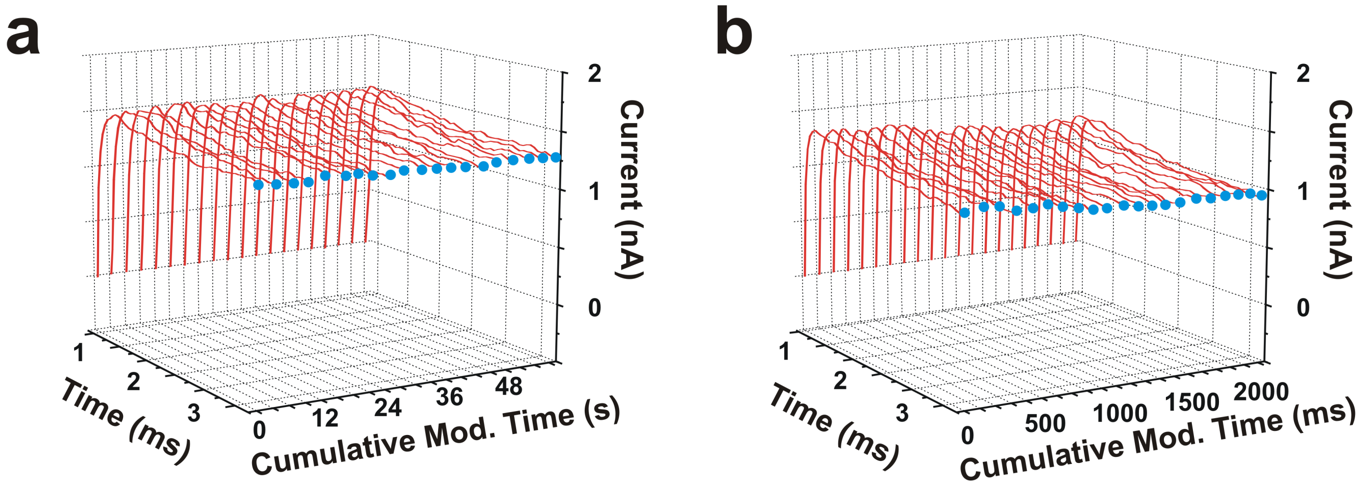

Supplementary Figure 1. MTSET application onto closed and open WSW channels does not reduce current amplitude. (a) Closed state modification experiment for WSW channels using 10 mM MTSET. After a total of 48 seconds exposure to MTSET, the initial and final current amplitude remain the same. (b) Open state experiment for WSW channels using 20 mM MTSET. After a cumulative MTSET exposure time of two seconds onto open WSW channels, no change in current amplitude is observed.

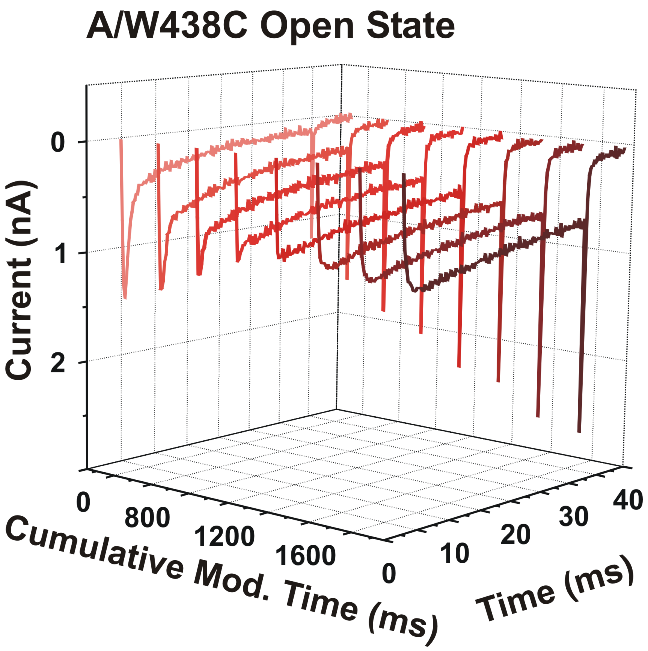

Supplementary Figure 2. Reaction time course of A/W438C with MTSET while channels are open. Initial currents (pink) recorded from inside-out patches containing A/W438C channels reveals that replacement of the tryptophan at this position with cysteine results in a near complete return of fast-inactivation. Repeated application of MTSET onto these channels while they are open (pink to dark red) progressively removes fast-inactivation and renders channels much like the WSW background. Of the three sites in the WSW mutant, this particular position is closest to the cytoplasm.
